# A novel indicator to predict the outcome of percutaneous stereotactic radiofrequency rhizotomy for trigeminal neuralgia patients: diffusivity metrics of MR-DTI

**DOI:** 10.1038/s41598-024-59828-4

**Published:** 2024-04-22

**Authors:** Xu Su, Zhengming Wang, Zhijia Wang, Min Cheng, Chao Du, Yu Tian

**Affiliations:** 1 Departments of Neurosurgery, The Third Hospital of Jilin University and China-Japan Union Hospital, 126 Xiantai Street, Changchun, 130033 Jilin People’s Republic of China; 2 Departments of Neurosurgery, Xinqiao Hospital, Army Medical University, Chongqing, 400037 People’s Republic of China; 3 Departments of Trauma Center, The Third Hospital of Jilin University and China‑Japan Union Hospital, Changchun, 130033 Jilin People’s Republic of China; 4 Departments of Radiation, The Third Hospital of Jilin University and China‑Japan Union Hospital, Changchun, 130033 Jilin People’s Republic of China

**Keywords:** Diffusion tensor imaging, Trigeminal neuralgia, Abnormal ganglion, Trigeminal ganglion, Percutaneous stereotactic radiofrequency rhizotomy, Neuroscience, Diseases of the nervous system

## Abstract

Magnetic resonance-diffusion tensor imaging (MR-DTI) has been used in the microvascular decompression and gamma knife radiosurgery in trigeminal neuralgia (TN) patients; however, use of percutaneous stereotactic radiofrequency rhizotomy (PSR) to target an abnormal trigeminal ganglion (ab-TG) is unreported. Fractional anisotropy (FA), mean and radial diffusivity (MD and RD, respectively), and axial diffusivity (AD) of the trigeminal nerve (CNV) were measured in 20 TN patients and 40 healthy control participants immediately post PSR, at 6-months, and at 1 year. Longitudinal alteration of the diffusivity metrics and any correlation with treatment effects, or prognoses, were analyzed. In the TN group, either low FA (value < 0.30) or a decreased range compared to the adjacent FA (dFA) > 17% defined an ab-TG. Two-to-three days post PSR, all 15 patients reported decreased pain scores with increased FA at the ab-TG (*P* < 0.001), but decreased MD and RD (*P* < 0.01 each). Treatment remained effective in 10 of 14 patients (71.4%) and 8 of 12 patients (66.7%) at the 6-month and 1-year follow-ups, respectively. In patients with ab-TGs, there was a significant difference in treatment outcomes between patients with low FA values (9 of 10; 90%) and patients with dFA (2 of 5; 40%) (*P* < 0.05). MR-DTI with diffusivity metrics correlated microstructural CNV abnormalities with PSR outcomes. Of all the diffusivity metrics, FA could be considered a novel objective quantitative indicator of treatment effects and a potential indicator of PSR effectiveness in TN patients.

## Introduction

Trigeminal neuralgia (TN), a severe neuropathic pain disorder, affects one or more branches of the trigeminal nerve (CNV, cranial nerve five) and occurs in 4–5 new patients per 100,000 annually^1,2^. Percutaneous stereotactic radiofrequency rhizotomy (PSR) is a minimally invasive therapy for TN, particularly when no neurovascular contact is observed on imaging^3,4^, and is often the primary treatment option for patients who are older, weaker, have had recurrence after failed microvascular decompression (MVD), or who are reluctant to undergo MVD^5,6^. A more quantitative neuroimaging technique, magnetic resonance-diffusion tensor imaging (MR-DTI), has been widely used in the study of TN^6–11^.

MR-DTI is based on the principle of the motion of water molecules in neural tissue. DTI has recently become widely used for the non-invasive delineation of nerve fiber tracts via tractography^12,13^ which can accurately delineate the visual 3D anatomical structures of nerve tracts and their branches, otherwise not observed on traditional morphological MRI^14,15^. A tract-specific DTI protocol indirectly assesses the microstructures of the nerve fiber, axon, and myelin^2,16^, thus providing a clearer visualization and estimation of the changes in the neural tract at the microscopic level, which is crucial in understanding the pathogenesis and treatment of TN^11,13,16,17^.

Recently, the DTI studies^9,18–20^ in patients with TN has focused on the root entry zone (REZ) of the CNV; however, no studies on abnormal trigeminal ganglion (ab-TG) have been reported. The aim of this was to investigate whether changes in diffusivity metrics during radiofrequency treatment in TN patients with ab-TG of TN could be correlated with outcomes.

## Materials and methods

### Study design and participants

This observational study involved 20 TN patients (TN group) and 40 healthy control participants. The TN group was further divided into an operative subgroup and a nonoperative subgroup. All patients underwent MR-DTI. The inclusion criteria for the TN group were as follows: (1) TN diagnosis according to the International Classification of Headache Disorders (3rd edition)^21^; (2) treatment between March 2021 and June 2022 in the Department of Neurosurgery at China-Japan Union Hospital of Jilin University (Changchun, Jilin, China). The inclusion criteria for the healthy control participants were as follows: (1) no prior medical record of neurological, psychiatric illnesses; (2) no experience of trigeminal nerve-related pain in the previous 6 months.

All study procedures were approved by the Institutional Review Board of China-Japan Union Hospital of Jilin University. All participants provided written informed consent. All methods were performed in accordance with the relevant guidelines and regulations.

### MR-DTI data acquisition, postprocessing, and analysis

Magnetic resonance-DTI images, including high-resolution T1-weighted 3D-sagittal anatomical images and DTI images, were acquired from a 3.0 Tesla Siemens MR scanner^18,22^. Anatomical images: in-plane voxel size = 0.9 × 0.9 mm^2^, slice thickness = 0.9 mm, 192 slices, matrix = 256 × 256, field of view = 24 × 24 cm^2^, echo time (TE) = 2.3 ms, repetition time (TR) = 2300 ms, and inversion time = 900 ms. DTI protocol: spin-echo EPI sequence, b value = 0 and 1000 s/mm^2^, 12 b0 Averages, 3 b1000 Averages, 72-direction, in-plane voxel size = 2 × 2 mm^2^, slice thickness of 2 mm with no gap, 30 continuous slices, matrix size = 256 × 256, field of view = 25 × 25 cm^2^, flip angle = 90°, TE = 95 ms, TR = 4100 ms. The integral parallel acquisition technique was used, and the acceleration factor was 2, which can decrease image distortion from susceptibility artifacts. A b value of 1000 s/mm^2^ was used with 3× image averaging and a b value of 0 s/mm^2^ was used with 12× image averaging. Diffusion tensor images were motion and eddy current corrected before image processing, with appropriate correction applied to the b-matrix.

Image postprocessing was performed in a 3D Slicer V4.11 software package utilities (http://www.slicer.org/). The process generated scalar maps of fractional anisotropy (FA), mean diffusivity (MD), radial diffusivity (RD), and axial diffusivity (AD). A tractography was generated from the DTI data, which entails postprocessing Digital Imaging and Communications in Medicine (DICOM) files. For each participant, fiber tracts of the CNV were generated from tractography of the region of interest seeds on both sides of the CNV in axial view FA maps^7,15^. Image reconstruction was used to characterize the major diffusion direction of the fiber and fiber tracking from the pons to a ganglion within Meckel's cave.

**Figure 1:**
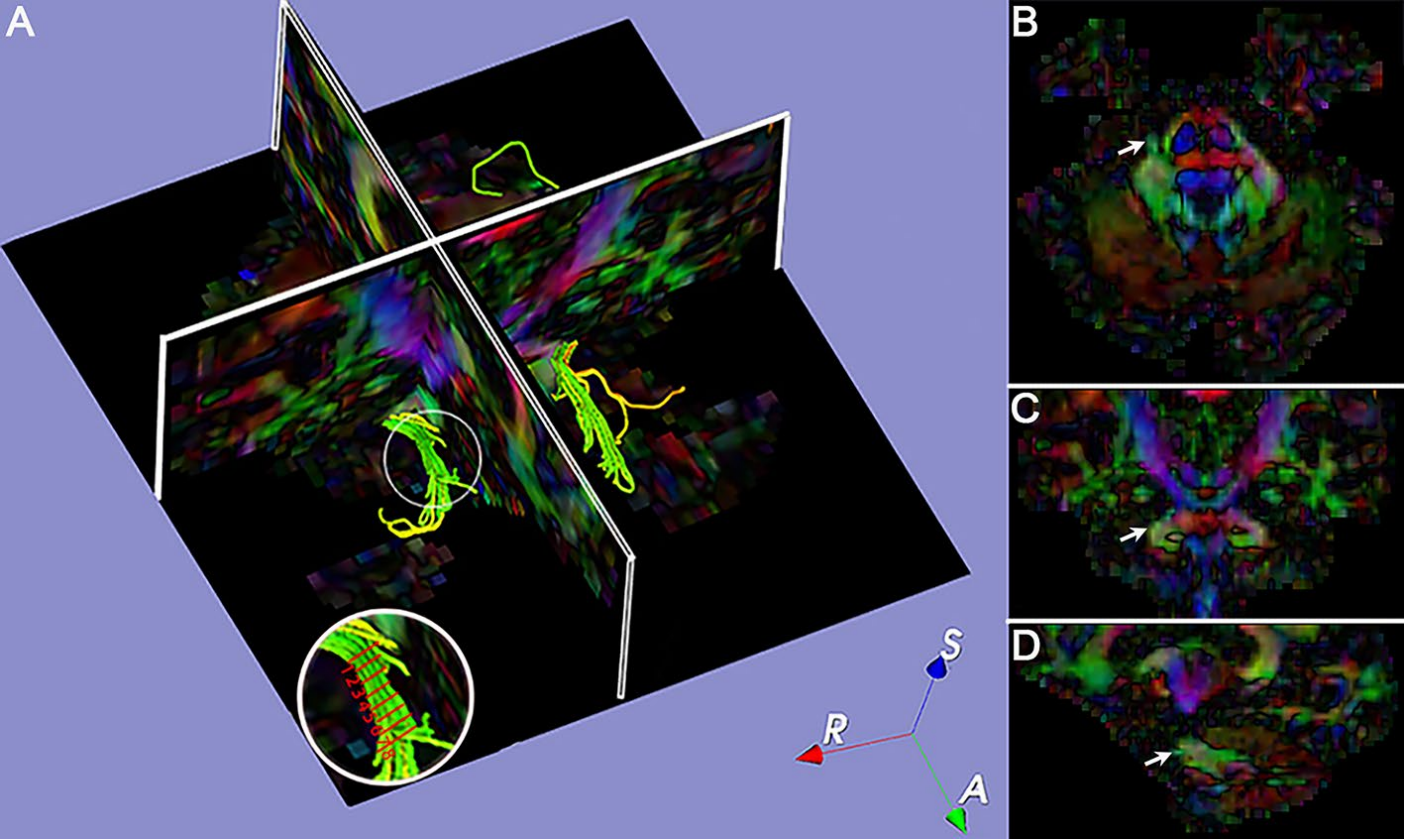
**Positioning and segmentation methods shown in fusion image of 3D-reconstruction CNV tract and color-fractional anisotropy maps.** Image (**A**) shows the segmentation of the CNV (white circle) in the axial image. Diffusion tensor images show the position (white arrow) of the CNV in the axial (**B**), coronal (**C**), and sagittal (**D**) views. R = right; S = superior; A = anterior; CNV = cranial nerve five (trigeminal).

**Figure 2:**
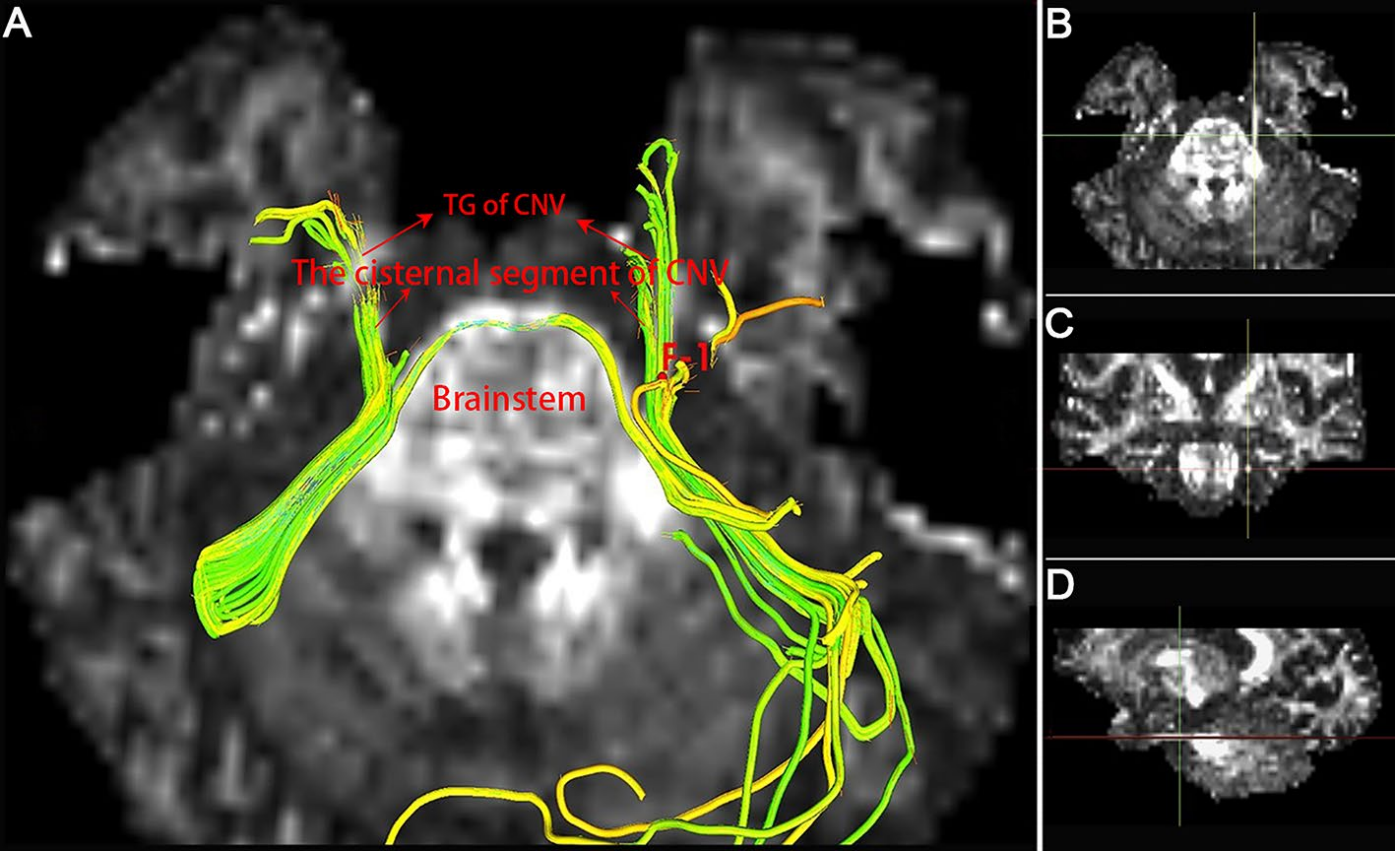
**Calculation of diffusivity metric values in diffusion tensor imaging. **Fusion image (**A**) of the 3D-CNV tracts and fractional anisotropy maps are used to distinguish the TG from the CNV tracts and for placing centroids (F-1) on tracts. Images (**B**, **C**, **D**) confirm the centroid position at the CNV coordinate intersection in the axial (**B**), coronal (**C**), and sagittal (**D**) views. CNV = cranial nerve five (trigeminal); TG = trigeminal ganglion.

The CNV tract was manually divided into eight segments (as Segs. 1–8) from the REZ in the pons to the TG in Meckel’s cavity according to the coronal DTI image (Fig. 1). Since the TG is crescent-shaped, the width of the TG ranged from 3.58 to 8.19 mm^23,24^. Thus, we selected four slices to extract diffusivity metrics of the TG (ie, Segs. 4–7. Seg. 1 was in the pons, Segs. 2–3 were in the REZ, and Seg. 8 was beyond the TG. To obtain diffusivity metrics (FA, MD, AD, and RD) of the CNV (Fig. 2), the centroid was placed in each tract segment on the successive coronal slices of scalar maps, then the position of the centroid was validated on the 3D planes and the CNV tracts^15,18^. In total, 1 to 3 DTI scans (preoperative, postoperative, and follow-up) were performed on patients in the TN group and one scan performed on participants in the control group.

### Detected abnormal segments of TGs in the analysis of diffusivity metrics of CNV

**Figure 3:**
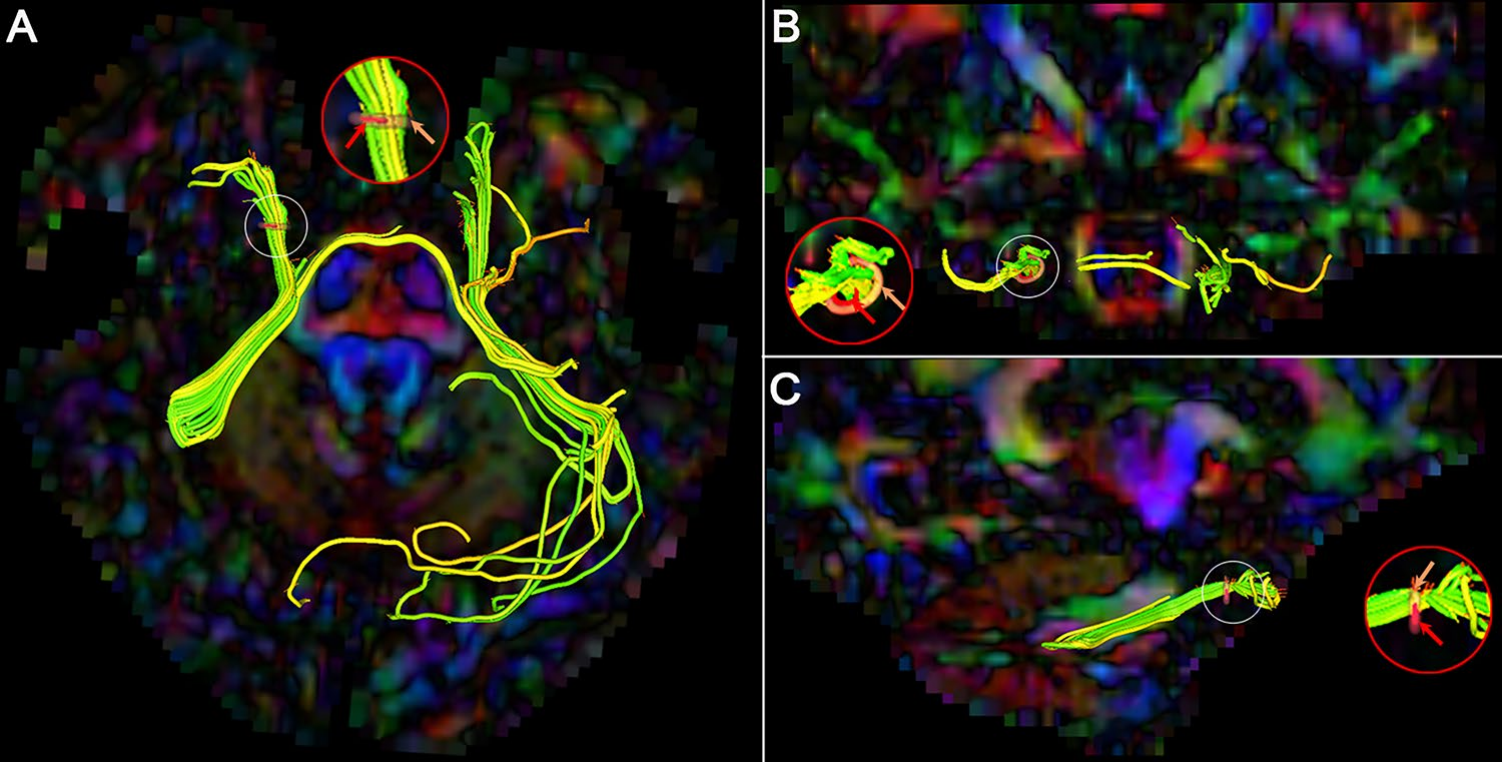
**Abnormal TGs in fusion images of 3D-reconstruction CNV and color-fractional anisotropy maps.** Image (**A**) confirms abnormal TG on CNV tracts in the axial view. Images (**B**, **C**) show the relationship between the location of the abnormal segment in TG (red arrows-internal loop) and the CNV tract (pink arrows-external loop) in the coronal (**B**) and sagittal (**C**) views. Inset red circle is an amplified image of the white circle. CNV = cranial nerve five (trigeminal); TG = trigeminal ganglion.

The trendline of diffusivity metrics was drawn according to the corresponding values of the eight CNV segments. The diffusivity metrics in DTI, such as FA and MD, were used to detect the abnormalities in the TG of TN patients. By analyzing diffusivity metrics in the TG segments in healthy control participants, the means ± standard deviations (SD) of the FA and a decreased range of FA (dFA) compared with the adjacent segment in controls were calculated and considered the reference for the abnormal FA threshold. The low limit of the FA on TG segments was 0.30 and the upper limit of the 95% confidence interval of the decreased range for TG segments was 16.39%. Therefore, the FA of an ab-TG was either < 0.30 or dFA > 17%. The means ± SDs of the MD, RD, and AD in the TG segments were also calculated. Abnormal TG segment characteristics such as position and size were measured in the diffusion-tensor images (Fig. 3).

### Classification

Trigeminal neuralgia can be categorized as classical TN (CTN), idiopathic TN (ITN), or secondary TN. Classical TN is the most common and is thought to be caused by neurovascular compression (NVC). The intensity of NVC can be divided into grade I (simple contact), grade II (a displacement or distortion), or grade III (a thinning nerve)^25–27^. According to the property of pain, TN is divided into Type I (paroxysmal pain) and Type II (continuous pain)^21^.

### Treatment

TN patients in the operative subgroup underwent PSR treatment. PSRs were performed using a stereotactic bidirectional approach-guiding technique^5,28,29^. The PSR was performed using Komai’s CT-Stereotactic Frame (Mizuho Medical Innovation https://www.mizuho.co.jp/en/neurosurgery/). 150 mm-long radiofrequency probe (diameter, 0.9 mm with 5-mm exposed tip) was used during PSR. During PSR operations, after fixing the stereotactic frame according to the 3D data of foramen ovale target (FOT) and the data of facial entry point, adjust the arc angle and the arm angle of stereotactic frame according to the characteristic of TGT.

Meanwhile, TN patients in the nonoperative subgroup received medical treatment (carbamazepine or oxcarbazepine).

### Treatment evaluation and follow-up

Treatment effects were evaluated postoperatively (2–3 days) and at follow-up (3–6 months and at 1 year postoperative) using visual analogue scale (VAS) scores and diffusivity metrics (FA, MD, RD, and AD). A 75% reduction of pain scores was used to distinguish effective from ineffective treatments^20,30^.

### Statistical Analysis

Summary statistics were computed for all DTI metric values. Differences between the control group and the TN group were determined using the* t*-test. Differences in the treatment effects and diffusivity metrics on TG were analyzed using the chi-square test. The DTI metrics were compared pre-and postoperatively in the TN group using the one-way repeated measures analysis of variance (ANOVA). If the assumption of “sphericity” is not satisfied, the F values can be corrected by the Greenhouse–Geisser method. All statistical analyses were performed using SPSS v26.0 (IBM, USA). In all statistical tests, *P* < 0.05 was considered statistically significant.

### Ethics approval and consent to participate

The study was approved by the Ethics Committee of the China-Japan Union Hospital of Jilin University. Written informed consent was obtained from the patient for the publication of this record and accompanying images.

## Results

### Patient characteristics

In the control group, ages ranged from 24 to 67 years and included 12 males and 28 females. In the TN group, the baseline characteristics of the 20 TN patients are summarized in Table 1. There were no significant differences in sexes or ages between the two groups. Fifteen patients in the TN operative subgroup had ab-TGs (Nos. 1–15). Five patients in the TN nonoperative subgroup selected medical treatment (Nos. 16–20).

**Table 1:** Characteristics of TN patients (n = 20).

Pt	Sex	Age	Affected divisions	Affected side	Pre-Tx	Post-Tx		Follow-up		NVCs grades
					VAS	VAS	VAS (%↓)	VAS	VAS (%↓)	
01	F	61	V2	R	10	2	80	1	90	CTN (I)
02	F	38	V1, V2, V3	R	10	1	90	0	100	CTN (II)
03	M	78	V1, V2	L	10	2	80	1	90	CTN (II)
04	F	85	V1	R	10	2	80	1	90	CTN (III)
05	M	65	V2, V3	R	10	3	70	2	80	CTN (I)
06	M	74	V1, V2	R	10	3	70	1	90	CTN (I)
07	F	71	V1, V2	L	10	1	90	0	100	CTN (II)
08	M	72	V2	R	10	4	60	3	70	CTN (III)
09	F	59	V2, V3	L	10	1	90	1	90	ITN
10	F	66	V1, V2, V3	R	10	4	60	3	70	CTN (III)
11	M	47	V3	R	10	1	90	3	70	CTN (II)
12	M	65	V2, V1	R	10	1	90	4	60	CTN (II)
13	M	65	V2	R	10	1	90	1	90	ITN
14	F	75	V1, V2, V3	L	10	1	90	1	90	CTN (I)
15	F	63	V2	R	10	1	90	1	90	ITN
16	M	75	V2	L	8	N/A				CTN (III)
17	F	51	V3	R	9	N/A				CTN (I)
18	M	48	V2	R	10	N/A				N/A
19	F	51	V3	R	3	N/A				N/A
20	F	59	V3	R	4	N/A				N/A

### CNV diffusivity metrics in healthy control participants

**Figure 4:**
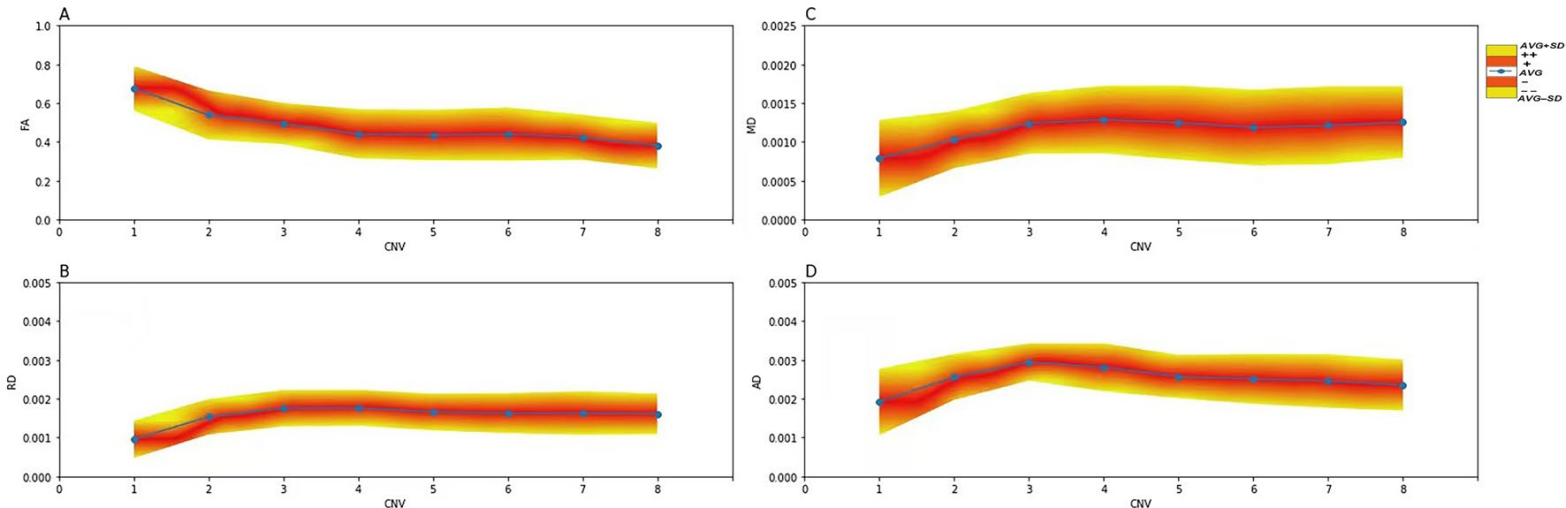
**Diffusivity metrics (FA, MD, AD, and RD) of CNV in healthy controls.** Image (**A**) shows that the average FA values are gradually decreasing along with the CNV segments. Images (**B, C** and** D**) show the average MD, AD, and RD values gradually increasing along with the CNV segments. Blue dot = average value; − Avg = minus 1–50%SD; − − Avg = minus > 50–100%SD; + Avg = plus 1–50%SD; +  + Avg = plus > 50–100%SD. AD = axial diffusivity; CNV = cranial nerve five (trigeminal); FA = fractional anisotropy; MD = mean diffusivity; RD = radial diffusivity.

Fractional anisotropy values gradually decreased from the pons to the TG; MD, AD, and RD values gradually increased from the pons to the TG. There were no major fluctuations along the CNV segments (Fig. 4). FA mean ± SD values in the TG segments were calculated (Table 2) and a lower limit of the normal range in the TG established at 0.30. The decreased ranges of FA values compared to adjacent TG segments were 10.66%, 1.44%, 1.18%, and 3.70% for Segs. 4, 5, 6, and 7, respectively. A 95% confidence interval of the decreased range for each segment as a reference for abnormal FA and the upper limit of dFA was established at 16.39%. Hence, either FA < 0.30 or dFA > 17% defined an ab-TG.

**Table 2:** The Mean ± SD of diffusivity metrics on TG in controls.

Mean ± SD	Seg.4	Seg.5	Seg.6	Seg.7
FA	0.44 ± 0.13	0.44 ± 0.13	0.44 ± 0.14	0.42 ± 0.12
MD	0.0018 ± 0.0005	0.0017 ± 0.0005	0.0016 ± 0.0005	0.0016 ± 0.0006
RD	0.0013 ± 0.0004	0.0012 ± 0.0005	0.0012 ± 0.0005	0.0012 ± 0.0005
AD	0.0028 ± 0.006	0.0026 ± 0.0006	0.0025 ± 0.0006	0.0025 ± 0.0007

The means ± SD of MD, RD, and AD on TG segments 4 through 7 were calculated (Table 2). Abnormal diffusivity metrics were determined as MD > 0.0022, RD > 0.0017, or AD > 0.0034.

### CNV diffusivity metrics in TN patients

In the TN group, 18 patients with VAS of 10 had ab-TGs. Two patients with VAS of 3 or 4 did not have ab-TGs. The rate of detecting ab-TGs was 90% (18/20) in TN patients, and the rate was 100% in patients with VAS of 10.

**Figure 5:**
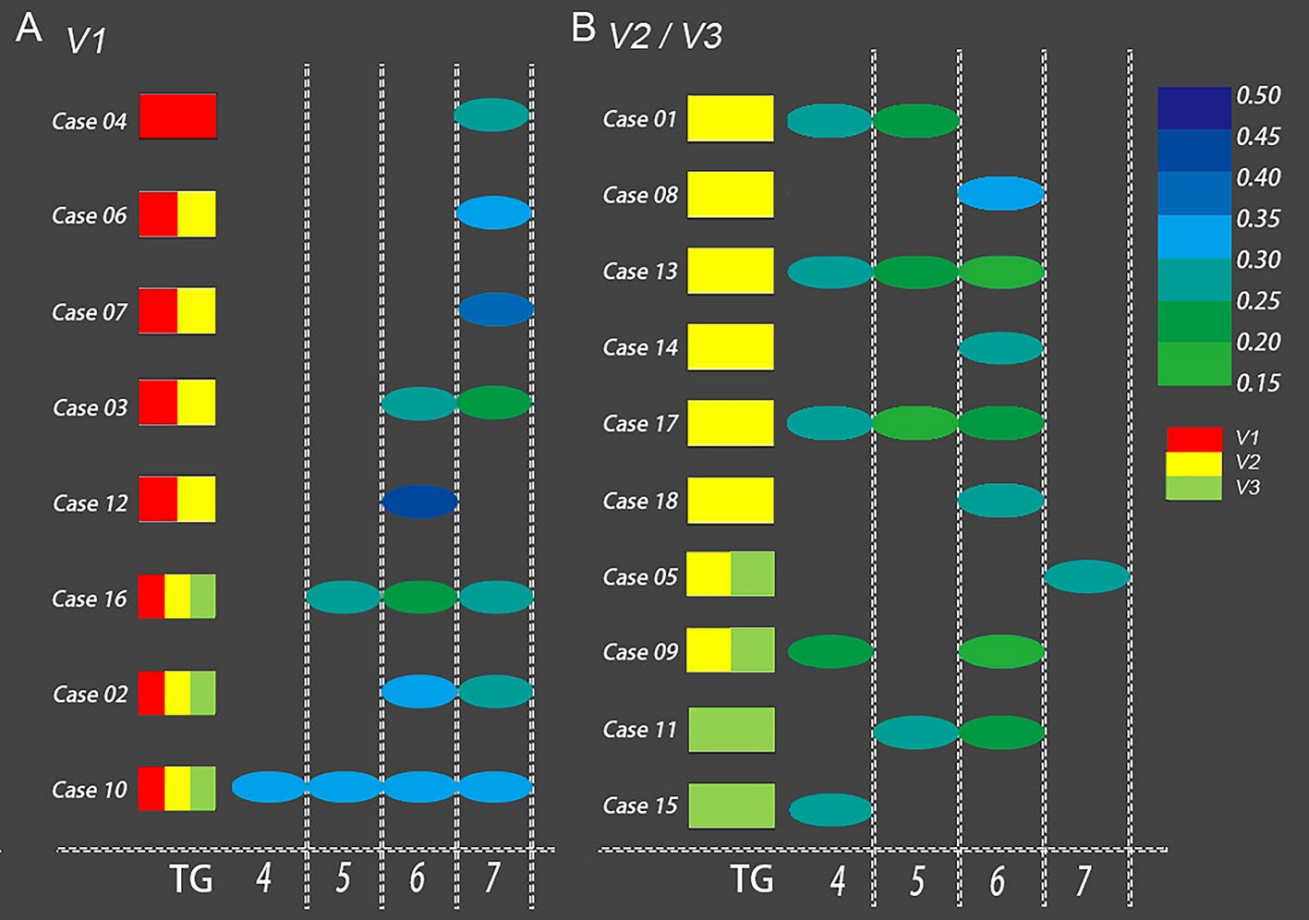
**Distribution of abnormal fractional anisotropy segments in 18 patients with TN. **Image (**A**) shows the abnormal segments with different FA values in the TG (Segs. 4–7) of the CNVs in V1-affected TN patients. The color intensity shifts from green to dark blue to indicate increasing FA values. Image (**B**) shows the abnormal segments with the different FA values in the TG (Segs. 4–7) of the CNVs in V2- or V3-affected TN patients. Red: V1; Yellow: V2; Green: V3. CNV = cranial nerve five (trigeminal); FA = fractional anisotropy; TG = trigeminal ganglion; TN = trigeminal neuralgia.

In the 18 patients with ab-TGs, a total of 30 affected branches and 32 abnormal segments were identified (Fig. 5). In the operative TN subgroup, ab-TGs were confirmed by low FA (< 0.30) in 10 patients (10/15, 66.7%) and by dFA > 17% (non-low FA) in another five patients (5/15, 33.3%). The distribution of the abnormal branches and abnormal FA segments in the TGs are shown in Fig. 5).

**Figure 6:**
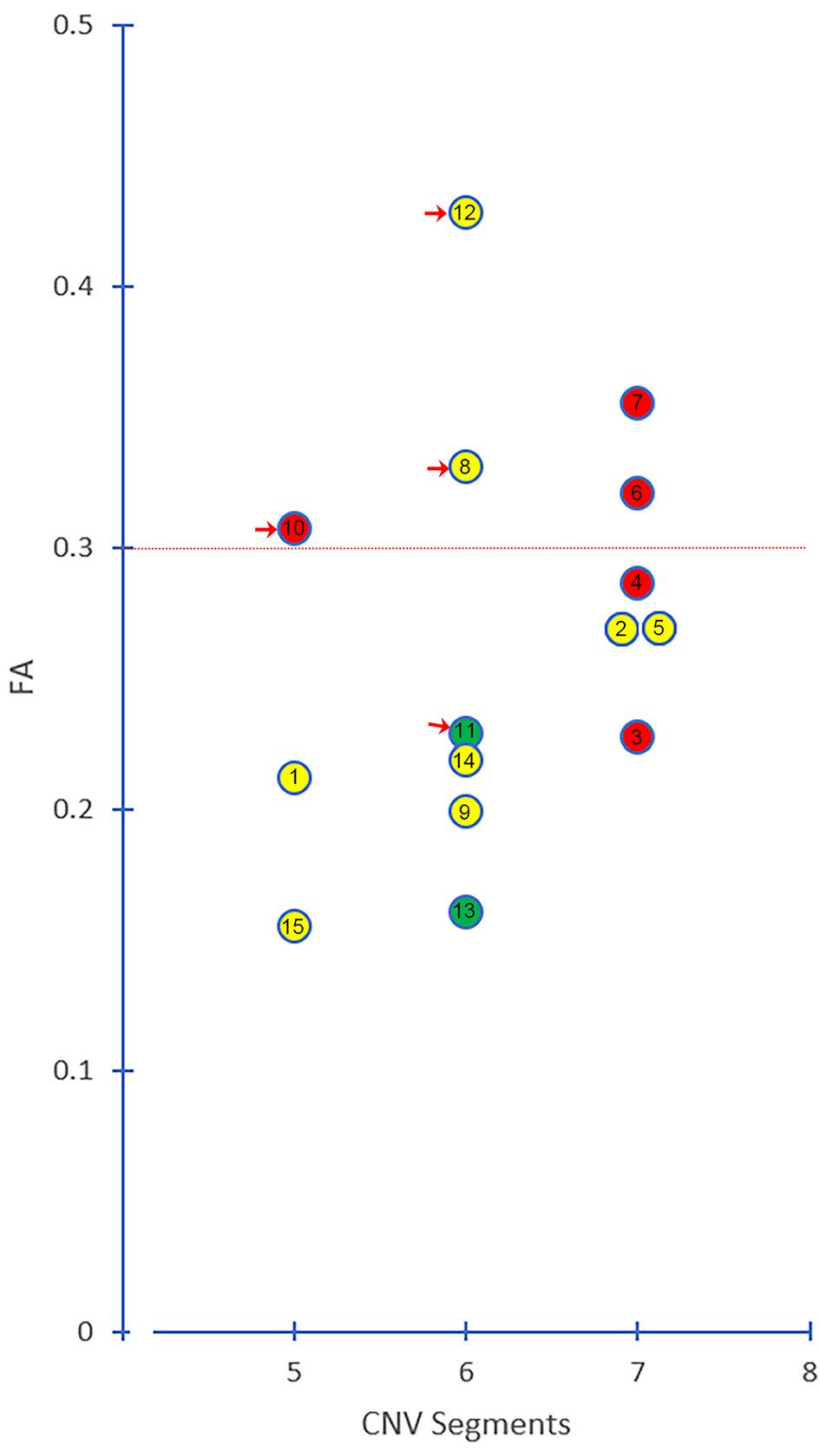
**Distribution of the trigeminal ganglion segments with the lowest fractional anisotropy value. **The graph shows the distribution of the lowest FA value in the trigeminal ganglion segments. The red dotted line indicates an FA level of 0.30. Each circle represents a patient and FA value. Red arrows indicate patients with ineffective treatment. Red circle = affected V1 segment; yellow circle = affected V2 segment; green circle = affected V3 segment. FA = fractional anisotropy.

The distribution of segments with the lowest FA in the ab-TG was as follows (Fig. 6): the abnormal TG segment in V1-affected patients was in Seg. 7 (80.0%, 4/5); in V2-affected patients was in Segs. 6 or Seg. 7 (75.0%, 6/8); and in V3-affected patients was in Seg. 6 (100.0%, 2/2).

**Figure 7:**
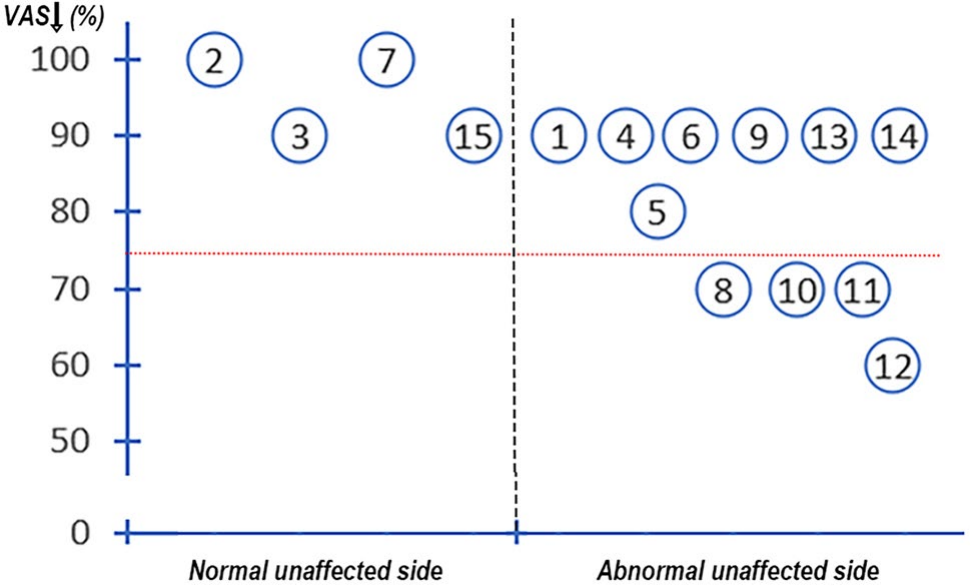
**The fractional anisotropy manifestation of the unaffected side of the CNV and the degree of pain relief (decreased VAS). **The graph shows the correlation between pain relief and the fractional anisotropy manifestation of the unaffected side of the CNV. The red dotted line indicates a 75% relief in pain as determined by a visual analogue scale.

The trendline of FA on the unaffected side of the CNV in four patients was accordant with the smooth trendline of FA in controls while 11 patients did not follow this pattern (Fig. 7, Table 3).

**Table 3:** The preoperative manifestation of FA in the CNV and treatment effects in TN patients (n = 15).

Pain scores ↓ (%)	The affected side of the CNV				The unaffected side of the CNV	
	REZ		TG		Normal	Abnormal
	Normal	Abnormal	Low	Non-low		
Effective	85.7% (6/7)	62.7% (5/8)	90% (9/10)	40% (2/5)	100% (4/4)	63.6% (7/11)
Ineffective	14.3% (1/7)	37.5% (3/8)	10% (1/10)	60% (3/5)	0	36.3% (4/11)

In the REZ of the affected CNV side, a sharp decrease in FA was noted 8 patients and no decrease in FA was noted in 7 patients (Table 3).

### PSR treatment

The success rate of single-puncture access was 93.3% (14/15) in TN patients who underwent PSR. During the procedure, the electrophysiology sensory test voltage ranged from 0.15 to 0.30 V.

### Operation efficacy and complications

**Figure 8:**
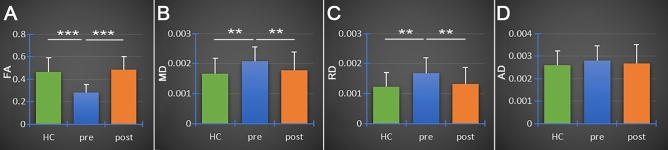
**Bar graphs showing diffusivity metrics (FA, MD, AD, and RD) in healthy controls and pre- and postoperatively in patients with TN. **The images show significant differences between healthy controls and TN patients in FA (*P* < 0.001***) (**A**), MD (*P* < 0.01**) (**B**), and RD (*P* < 0.01**) (**C**) values. There were no significant differences observed between healthy controls and TN patients in AD values (*P* > 0.05) (**D**). Green bar = healthy controls; blue bar = preoperative TN group; orange bar = postoperative TN group. AD = axial diffusivity; FA = fractional anisotropy; MD = mean diffusivity; RD = radial diffusivity.

The immediate effects of PSR were evaluated 2–3 days postoperatively. VAS decreased postoperatively to 1–3 in 13 patients (one patient with VAS of 3 and 12 patients with VAS of 1) and to 4 in 2 patients. The postoperative VAS decrease ranged from 60 to 90% in all patients. Postoperative DTI scans were performed on 14 patients (one patient had no postoperative DTI scan). After radiofrequency treatment of the TG, the postoperative FA of the abnormal TG segment had increased by 14.1% in 1 patient (No. 8), 33.8–81.8% in 9 patients, and 124.2–274.9% in 4 patients. There were significant differences between the preoperative and postoperative FA (*P* < 0.001), MD (*P* < 0.01), and RD (*P* < 0.01) values in the abnormal TG segments, but not in AD values (*P* > 0.05) (Fig. 8). On the unaffected CNV side, diffusivity metrics between preoperative and postoperative illustrated no significant differences (Table 4) suggesting that preoperative FA, MD, and RD values changed significantly after PSR in patients with ab-TGs.

**Table 4:** Diffusivity metrics of the unaffected side in pre-and postoperative (n = 14).

Unaffected side	FA		MD		RD		AD	
	Pre	Post	Pre	Post	Pre	Post	Pre	Post
Mean	0.5265	0.4794	0.0016	0.0017	0.0015	0.0015	0.0023	0.0023
Median	0.5063	0.4080	0.0016	0.0017	0.0015	0.0015	0.0024	0.0025
SD	0.1777	0.1425	0.0005	0.0004	0.0006	0.0007	0.0008	0.0009
*T*-test	*P* > 0.05		*P* > 0.05		*P* > 0.05		*P* > 0.05	

By analyzing diffusivity metrics and VAS preoperatively and postoperatively, FA and MD values were synchronously changed in detecting the abnormal CNV segment and the diffusivity metrics (FA, MD) and the VAS changed simultaneously. The changes in direction of the FA and MD values were inverted in DTI. After treatment, the changes in FA in all patients (100%, 14/14) and MD in 78.6% of patients (11/14) were consistent with changes in VAS.

**Figure 9:**
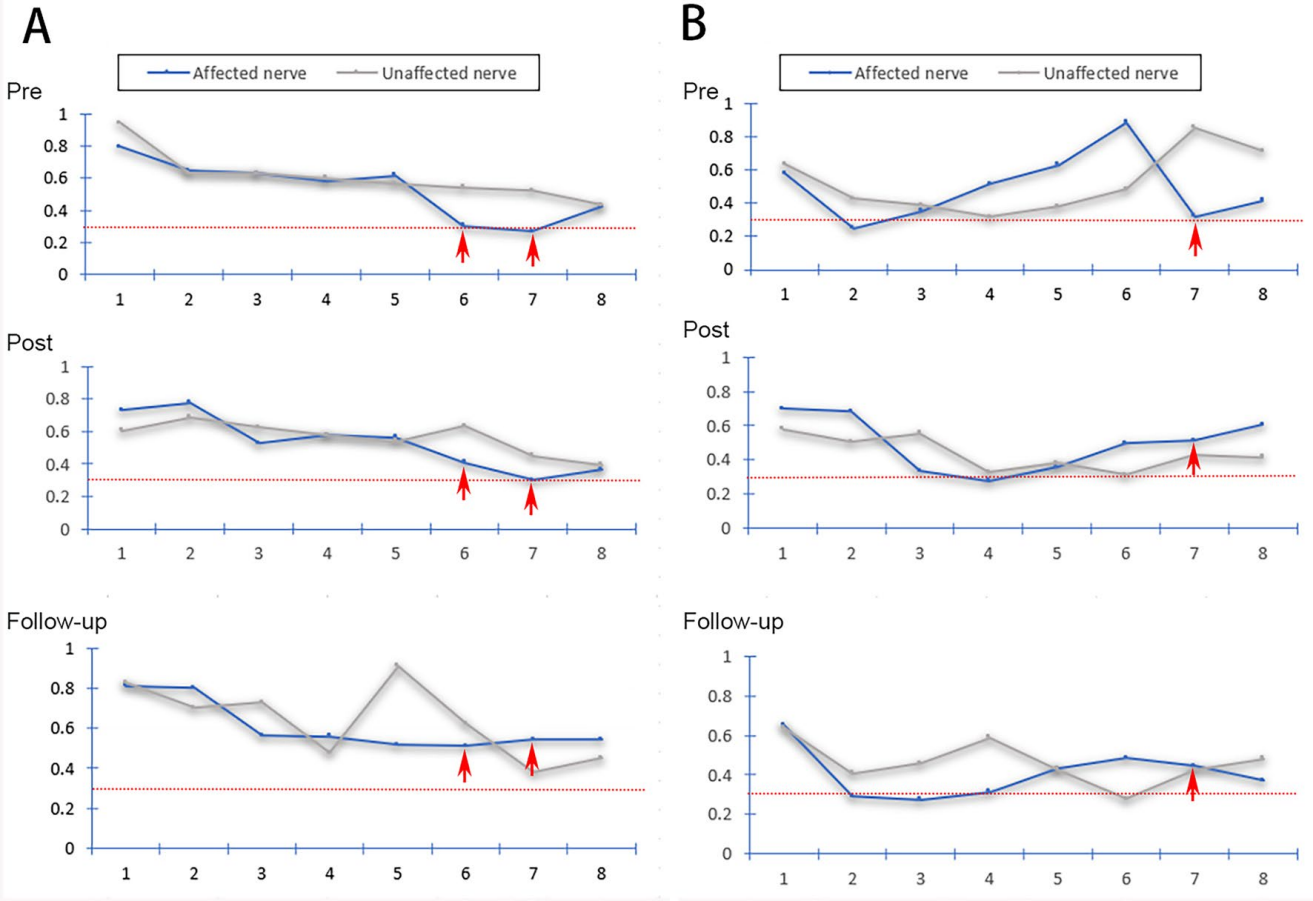
**Fractional anisotropy of the CNVs of two TN patients with effective treatments.** Diffusivity metrics (FA) of the CNVs of two TN patients (**A** and **B**) with effective treatments shown preoperatively (pre), 2–3 days postoperative (post), and at 3- to 6-month follow-up visits (follow-up). Image (**A**) shows the fractional anisotropy trendline of two sides of the CNV in patients with low FA in the TG segment. Image (**B**) shows the fractional anisotropy trendline of two sides of the CNV in patients with non-low FA in the TG segment. The red dotted line indicates an FA level of 0.30; red arrows: abnormal FA segments of the trigeminal ganglion.

**Figure 10:**
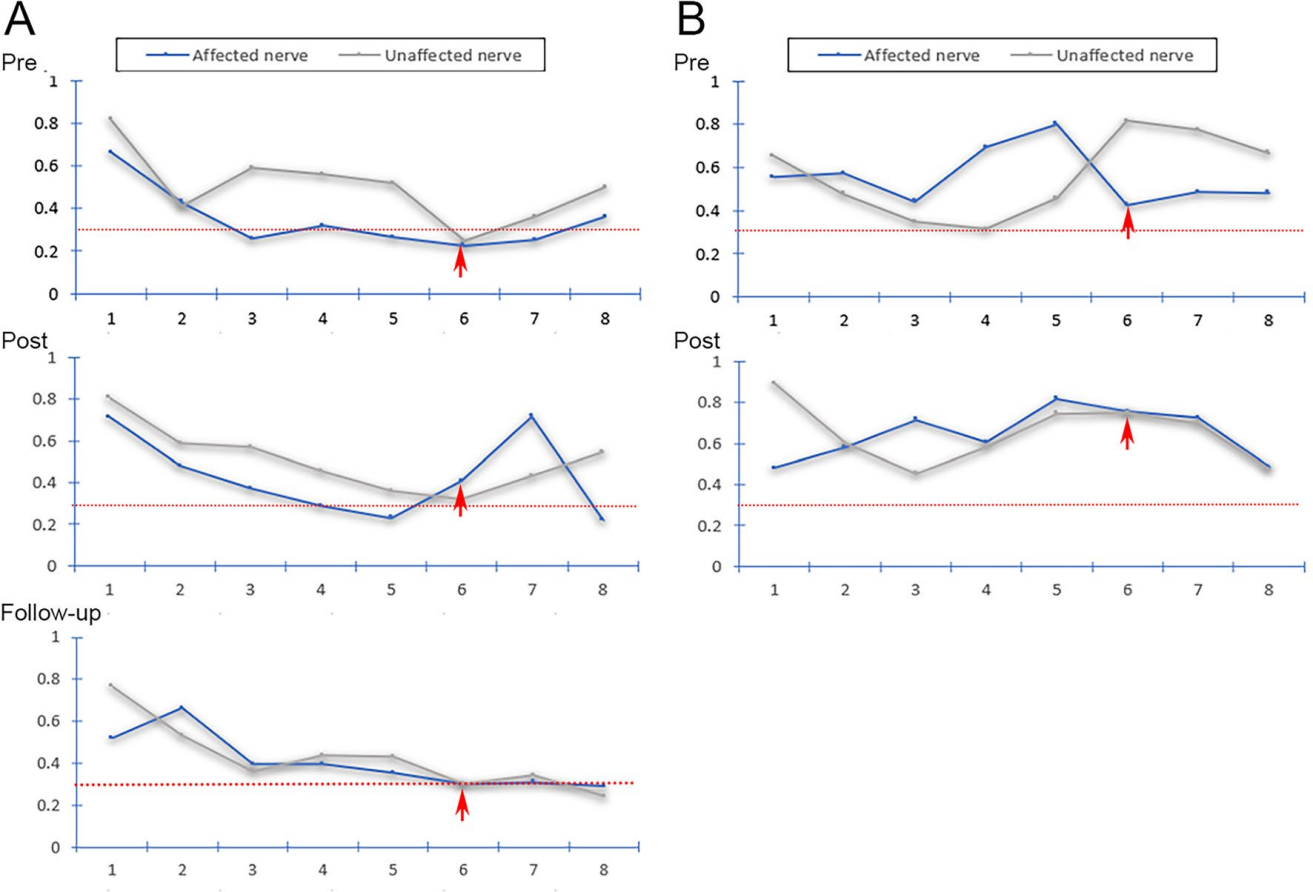
**Fractional anisotropy of the CNVs of two TN patients with ineffective treatments.** Diffusivity metrics (FA) of the CNV of two TN patients (**A** and** B**) with ineffective treatments shown preoperatively (pre), 2–3 days postoperative (post), and at 3- to 6-month follow-up visits (follow-up). Image (**A**) shows the fractional anisotropy trendline of two sides of the CNV in patients with low FA in the TG segment. Image (B) shows the fractional anisotropy trendline of two sides of the CNV in patients with FA > 0.30 in the TG segment. The red dotted line indicates an FA level of 0.30; red arrows = abnormal FA segments of the trigeminal ganglion.

Fifteen patients who underwent PSR had follow-up evaluations at the 3- to 6-month postoperative time period; 14 of these patients were evaluated at the 6-month mark. Five patients underwent DTI at the 3- to 6-month follow-up. VAS was 0–2 in 10 patients and 3–4 in 4 patients (No. 8, 10, 11 and 12). Treatment was effective in 11 patients (11/15, 73.3%) (Fig. 9) and ineffective in 4 patients (Fig. 10). Of the 11 patients with effective treatments, 9 patients (81.8%) had low FA values (i.e., < 0.30) and 2 patients (18.2%) had FA values ≥ 0.30 in the TG. Of the 4 patients classified as having ineffective treatment, small focal areas of recurrent pain were noted including 3 patients (Nos. 8, 10 and 12) with FA values ≥ 0.30 and 1 patient (No. 11) with an FA value < 0.30 in the TG. No patient experienced recurrence of the whole affected area.

In 5 patients who underwent DTI at the 3- to 6-month follow-up period, 4 patients with effective treatment had further FA increases on TG segments that ranged from 24.7 to 103.8% when compared to their preoperative values. One patient with ineffective treatment experienced a decrease in their FA value on TG segments by 26.6% when compared to their 3-day postoperative FA value.

**Figure 11:**
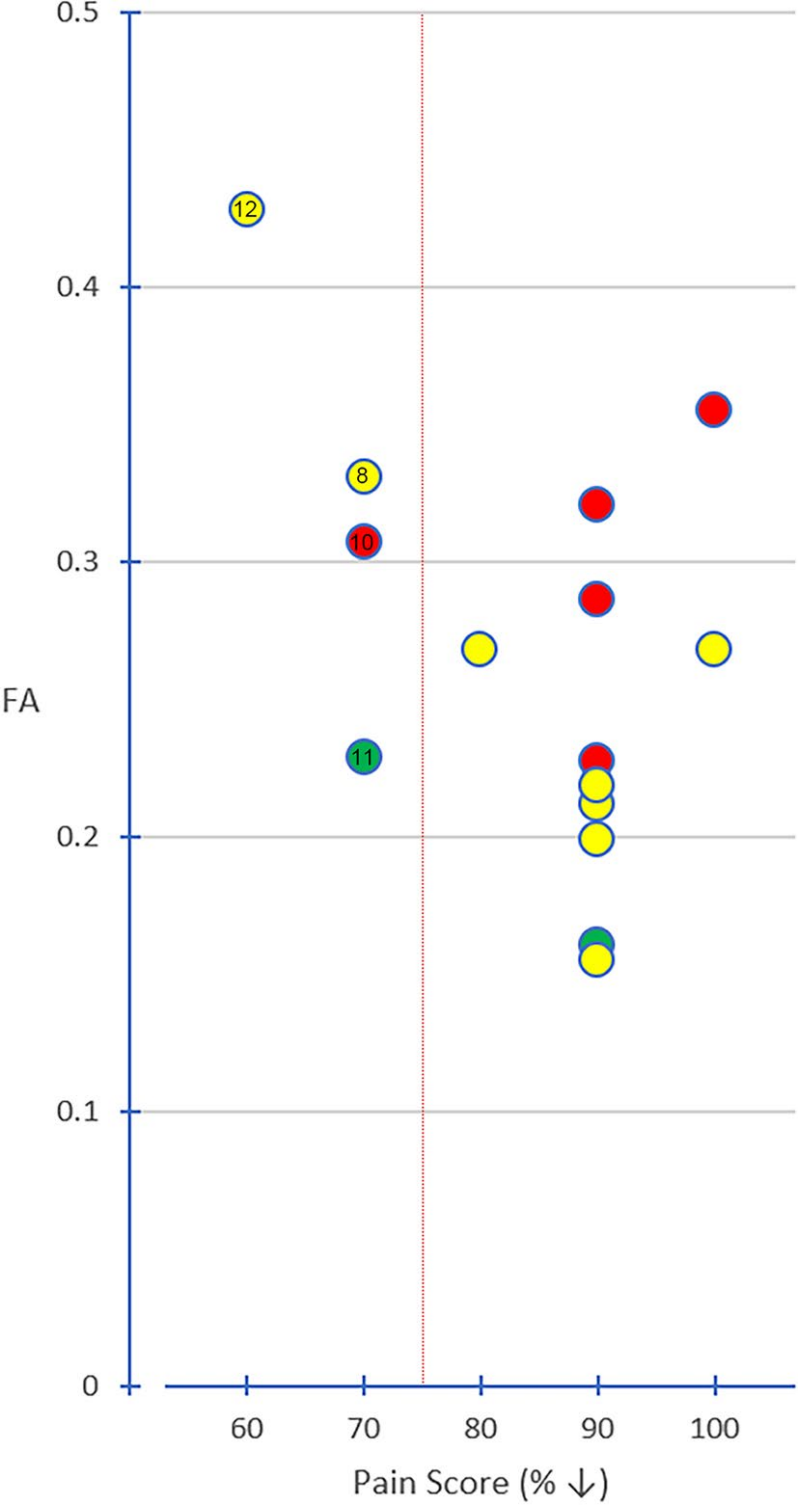
**The correlation between the lowest FA values in TG and pain relief.** The graph shows the correlation between the lowest fractional anisotropy values in the trigeminal ganglion and pain relief after treatment. The red dotted line indicates a 75% relief in pain as determined by a visual analogue scale. Each circle with a number represents a patient with ineffective treatment and their FA value. Red circle = affected V1 segment; yellow circle = affected V2 segment; green circle = affected V3 segment.

Treatment outcomes could be correlated with the TG segments in patients with the lowest FA values (Fig. 11). Of the patients with low FA in the affected side of the TG, nine patients experienced effective treatments (9/10, 90%) and treatment was ineffective in one patient (No. 11) (Table 3, Fig. 11). Of the patients with FA ≥ 0.30 in the affected side of the TG, 2 patients experienced effective treatments (2/5, 40%) and treatment was ineffective in 3 patients (3/5, 60%; No. 8, 10, 12). The chi-square test was used for comparing the differences between the lowest FA values and the treatment outcomes (chi-square value: 4.261, *P* < 0.05).

The FA manifestations of the affected REZ were normal in 7 patients and abnormal in 8 patients at the preoperative evaluation (Table 3). Of the 7 patients with normal FA manifestations, treatment was effective in 6 patients (6/7, 86%) and ineffective in one patient. Of the 8 patients with abnormal FA manifestations, treatment was effective in 5 patients (5/8, 62.5%) and ineffective in 3 patients (3/8, 37.5%).

The FA manifestations of the unaffected CNV were normal in 4 patients and abnormal in 11 patients at the preoperative evaluation (Fig. 7, Table 3). Of the 4 patients with normal FA manifestations of the unaffected side CNV, treatment was effective in all patients (4/4, 100%). Of the 11 patients with abnormal FA manifestations of the unaffected side CNV, treatment was effective in 7 patients (7/11, 63.6%) and ineffective in 4 patients (4/11, 36.4%).

Diffusivity metrics in each of the 4 patients (Patient Nos. 8, 10, 11 and 12) with ineffective treatment noted at the 3- to 6-month postoperative period revealed the following: Patient No. 8 had low FA on the affected TG and an abnormal unaffected side of the CNV; Patient No. 10 had an FA value ≥ 0.30 on the affected TG, decreased FA on the REZ of the affected side CNV, and an abnormal unaffected side of the CNV; Patient No. 11 had an FA value ≥ 0.30 on the affected TG and an abnormal FA value of the unaffected side CNV; and Patient No. 12 had an FA value ≥ 0.30 on the affected TG and decreased FA on the REZ of the affected side CNV. Overall, 3 patients (75%, 3/4) had FA values ≥ 0.30 on the affected TG and 4 patients (100%) had decreased FA values on the REZ of the affected side CNV and/or an abnormal unaffected side of the CNV.

Twelve patients completed the 1-year follow-up. Eight patients reported effective treatments (VAS was 0–1), and the rate of effective treatment was 66.7% (8/12). In all 4 patients who had small focal areas of recurrent pain, 3 patients opted for medicinal treatment and one patient underwent MVD.

After treatment, facial numbness was noted in one patient (No. 1). None of most common complications were observed; however, Patient No. 10 experienced a rare complication of temporary apnea.

## Discussion

Diffusion tensor imaging, a technique based on the principle of diffusion motion of water protons in nerve tissue, has recently received attention in TN studies. Several studies support the use of DTI in TN for its ability to expose the CNV at a microstructure level^8,15,19,31–37^. DTI has been used to evaluate the efficacy of MVD and gamma knife radiosurgery (GKRS) with the REZ for TN as the therapeutic target^12,20,22^. In addition, DTI is also used to detect diffusivity metrics on the CNV^38^ or the REZ of the CNV^19^ both before and after radiofrequency treatment. To date, DTI has not been used to detect the abnormal diffusion on the TG in patients with TN or to guide surgical treatment targeting. Measuring the preoperative FA of the CNV can serve as a comparator post-treatment to evaluate the efficacy of PSR.

Fractional anisotropy is the most widely used quantitative metric to comprehensively reflect the diffusion profile in the dominant diffusion direction. Because of its high sensitivity to the microstructure of myelinated axons and neural injury^13,39,40^, FA has been even regarded as a prognostic biomarker of MVD and GKRS outcomes^9,22,41^. The MD and RD change synchronously with the FA; however, abnormalities of nerves might be amplified and reflected in FA. The authors determined that an abnormal TG could be defined by both a low FA (< 0.30) or a sharp FA decrease (> 17%) and that the abnormal TG and the lowest FA segments in TN patients showed a regular distribution. David et al. reported that the TG can be divided into 3 subregions corresponding to its 3 branches^42^. Current study results further corroborate the CNV and TG anatomical structures suggesting the utility of diffusivity metrics as an independently reliable method of detecting the abnormal segments of the TG.

DTI was used to evaluate the efficacy^9,20,22,41,43^ of MVD and GKRS with the therapeutic target in REZ for TN. Diffusivity metrics, especially FA, reflect the treatment efficacy of TN patients in the nerve microstructure ^19^. FA is generally regarded as a quantitative, noninvasive prognostic and pathology marker of TN^9,19,20,34,38–41,43^. The significant change in diffusivity on TG segments induced by PSR and the change in FA in all patients (100%, 14/14) and MD in 78.6% of patients (11/14) were consistent with changes in VAS. Current study results showed that FA and MD values in TN patients with abnormal TG could be useful indicators in objectively evaluating PSR efficacy.

In the study patients who underwent PSR, all 15 observed immediate efficacies which indicated that the FA of MR-DTI plays a crucial role in guiding the TG treatment target in PSR. At the 6-month follow-up visit, 4 of 15 patients (26.7%) reported ineffective treatment including 3 of 4 patients (75%) with ab-TGs identified by FA values ≥ 0.30 and one patient with an FA value < 0.30. Recurrence shortly following radiofrequency thermocoagulation (RFT) is a known issue. Several studies report recurrence rates of approximately 20–25% 6 months following RFT ^4,44–46^. We hypothesize that the primary reason for recurrence shortly after PSR may be due to an FA value ≥ 0.30 in the TG. It seems that the degree to which the FA value in the TG is abnormal portends the effectiveness of PSR. TN patients with FA values ≥ 0.30 in the TG may be more prone to recurrence post PSR or may not be candidates for PSR at all.

It was important to try and analyze whether FA values on every segment of the CNV could portend recurrence shortly after radiofrequency treatment. TN is characterized not only by diffusion abnormalities on the CNV when compared to controls, but also by abnormalities between the affected and unaffected sides of the TG ^18,31,39^. By combining diffusivity metrics of the bilateral CNVs, the current study results suggest the following findings may herald effective PSR treatment: normal FA value of the unaffected side of the CNV (4/4, 100%); an FA value < 0.30 in the affected side of the TG (9/10, 90%); and a normal FA value of the affected side REZ (6/7, 86%). Patients with FA values ≥ 0.30 in the affected side of the TG (3/5, 60%), an abnormal FA value of the affected side REZ (3/8, 38%), and an abnormal FA value of the unaffected side of the CNV (4/11, 36%) typically see poorer results following PSR. Diffusivity metrics of the bilateral CNVs may be indicators to predict the effect of PSR and detecting preoperative FA values in bilateral CNVs may improve PSR efficacy rates.

The rate of effective treatment was 66.7% (8 of 12 patients) at the 1-year follow-up. Recurrences were noted in 4 four patients and were limited to small areas of focal pain. Because the recurrences occurred so shortly after PSR (i.e., within 3–5 months), the authors had the opportunity to investigate potential reasons for suboptimal efficacy.

The most common complications of radiofrequency treatment include hematoma, facial swelling, facial hypoesthesia, masticatory weakness, meningitis, and cerebrospinal fluid leakage ^1,3–5^. One of our study patients experienced facial hypoesthesia and another patient developed a rare complication of temporary apnea related to vertebrobasilar dolichoectasia^6^. Overall, the use of MR-DTI-guided PSR resulted in a high single-puncture success rate (93.3%, 14/15) on the TG treatment target which likely contributed to reduced potential for subsequent complications.

The study did have its limitations. A more reliable criterion for abnormal CNV needs to be established in larger studies. Expanding the sample size will help us explore whether FA can be a potential indicator for future TN molecular typing diagnoses. In addition, the applicability of abnormal TGs in patients with TN needs longer follow-up. Extending the follow-up will confirm whether adjusting the RFT dose can improve the efficacy rate in patients with dFA and may eventually expand the indications for use of RFT.

## Conclusions

Microstructural abnormalities of CNV are associated with a spectrum of treatment effect in patients with TN. Utilization of MR-DTI with diffusivity metrics reveals microstructural alterations of the CNV and correlates with outcomes after PSR. Fractional anisotropy, in particular, could be considered a novel objective quantitative biomarker to assess treatment efficacy as well as a potential indicator of patient prognosis following PSR.

## Data Availability

The datasets used and/or analysed during the current study are available from the corresponding author on reasonable request.

## Abbreviations

ab-TG: Abnormal trigeminal ganglion
AD: Axial diffusivity
CNV: Cranial nerve five
CTN: Classical TN
dFA: A decrease range compared with the adjacent segment of FA
FA: Fractional anisotropy
GKRS: Gamma knife radiosurgery
ITN: Idiopathic TN
MD: Mean diffusivity
MR-DTI: Magnetic resonance-diffusion tensor imaging
MVD: Microvascular decompression
NVC: Neurovascular compression
RD: Radial diffusivity
REZ: Root entry zone
RFT: Radiofrequency thermocoagulation
PSR: Percutaneous stereotactic radiofrequency rhizotomy
SD: Standard deviation
TE: Echo time
TG: Trigeminal ganglion
TN: Trigeminal neuralgia
TR: Repetition time
VAS: Visual analogue score
